# The *Shigella* Type III Secretion System: An Overview from Top to Bottom

**DOI:** 10.3390/microorganisms9020451

**Published:** 2021-02-22

**Authors:** Meenakumari Muthuramalingam, Sean K. Whittier, Wendy L. Picking, William D. Picking

**Affiliations:** Department of Pharmaceutical Chemistry, University of Kansas, Lawrence, KS 66049, USA; mmeena85@ku.edu (M.M.); seanw@ku.edu (S.K.W.); wendy.picking@ku.edu (W.L.P.)

**Keywords:** *Shigella*, type III secretion system, T3SS, injectisome, tip complex, translocator, sorting platform

## Abstract

*Shigella* comprises four species of human-restricted pathogens causing bacillary dysentery. While *Shigella* possesses multiple genetic loci contributing to virulence, a type III secretion system (T3SS) is its primary virulence factor. The *Shigella* T3SS nanomachine consists of four major assemblies: the cytoplasmic sorting platform; the envelope-spanning core/basal body; an exposed needle; and a needle-associated tip complex with associated translocon that is inserted into host cell membranes. The initial subversion of host cell activities is carried out by the effector functions of the invasion plasmid antigen (Ipa) translocator proteins, with the cell ultimately being controlled by dedicated effector proteins that are injected into the host cytoplasm though the translocon. Much of the information now available on the T3SS injectisome has been accumulated through collective studies on the T3SS from three systems, those of *Shigella flexneri*, *Salmonella typhimurium* and *Yersinia enterocolitica*/*Yersinia pestis*. In this review, we will touch upon the important features of the T3SS injectisome that have come to light because of research in the *Shigella* and closely related systems. We will also briefly highlight some of the strategies being considered to target the *Shigella* T3SS for disease prevention.

## 1. Introduction

Many important Gram-negative bacterial pathogens of plants and animals use type III secretion systems (T3SS) to initiate contact with target eukaryotic cells to manipulate those cells for the benefit of the pathogen [1]. While largely restricted to communication with eukaryotic targets, T3SSs have a strong evolutionary relationship with the Gram-negative flagellum [2], which at its core uses the flagellar-T3SS (fT3SS) as a secretion apparatus, in part to assemble the extracellular flagellar components. Across many pathogens, the virulence T3SS apparatus or injectisome is architecturally well conserved with regard to its cytoplasmic, core and extracellular assemblies [3,4,5,6,7]. Much of the detailed information that is now available on the T3SS injectisome has been generated through detailed studies on the T3SS injectisome of species belonging to three bacterial genera—*Shigella flexneri*, *Salmonella typhimurium* and *Yersinia enterocolitica*/*Yersinia pestis*. This review will focus on some of the facets of the *Shigella* T3SS and injectisome. Included will be a discussion of how the *Shigella* system has improved our overall understanding of these systems and how it may be targeted for improving public health.

## 2. A Brief Overview of *Shigella*

The genus *Shigella*, an important pathovar of *Escherichia coli*, is generally considered to comprise four species (*S. flexneri*, *S. sonnei*, *S. dysenteriae* and *S. boydii*) with, collectively, more than 50 O-antigen serotypes [8,9]. *S. flexneri* causes moderate to severe diarrheal and dysenteric disease in much of the developing world where it is responsible for endemic cases of shigellosis [10,11]. *S. flexneri* is particularly problematic in wide swaths of southern Asia and on the African continent [12]. In contrast, *S. sonnei* is more often associated with outbreaks in higher income nations with outbreaks often associated with child daycare centers and other institutional settings such as schools or adult care centers [13,14]. Globally, *Shigella* is among the top four leading causes of moderate to severe infectious diarrhea in children—together with rotovirus, Enterotoxigenic *E. coli*, and *Cryptosporidium* [11]. While once reportedly being responsible for upwards of 1.1 M deaths annually in the 1990s [15], *Shigella* is now estimated to be responsible for about 200,000 deaths/year [10] and possibly even fewer [16]. While mortality has decreased over time, morbidity remains high along with extraintestinal complications caused by repeated episodes of moderate to severe diarrhea and dysentery. These manifestations can include malnutrition, stunted growth and impaired cognitive development [17].

Following ingestion, *Shigella* makes its way quickly to the large intestine, possibly with assistance from the Sen and Set enterotoxins [18,19,20]. It then initiates disease onset by crossing M-cells in the large intestine to gain access to the submucosa [21]. The initial contact with the macrophages results in phagocyte killing by a pyroptotic process that is accompanied by release of IL-1β [22]. This frees the pathogen beneath the colonic epithelium while the IL-1β initiates the onset of subsequent inflammatory events. The inflammation that ensues results in the recruitment of neutrophils that further contribute to bacterial infiltration of the epithelial layer and the localized severe inflammation and ulceration that gives rise to the symptoms of bacillary dysentery [23]. Meanwhile, the bacteria beneath the colonic epithelium require their T3SS to mediate contact at the basolateral sides of the overlying epithelial cells and inject the early effector proteins that promote membrane ruffling and bacterial entry. Internalized *Shigella* then escapes the pathogen-containing vacuole to take up residence in the cytoplasm where bacterial proliferation begins [24]. Subsequent dedicated T3SS effector proteins then work toward restoring normal cellular behavior, thereby contributing to the creation of a protective niche for bacterial growth [24,25]. Once in the cytoplasm, *Shigella* is able to subvert the host actin cytoskeleton in a T3SS-independent manner (via the polar-localized intercellular spread protein IcsA/VirG) to provide actin-based motility and eventual intercellular spread into neighboring cells [26,27].

*Shigella* are largely human-restricted pathogens that possess multiple genetic loci that contribute to their ability to cause disease; however, a functional T3SS is an essential component of this pathogen’s virulence factor arsenal [18]. The *Shigella* T3SS possesses a cytoplasmic sorting platform (SP) that powers secretion and is involved in effector protein selection [28]. The SP is loosely associated with the envelope-spanning core or basal body that provides substrate passage across the inner and outer bacterial membranes [29]. Extending outward from this core on the bacterial surface is a needle [30] that is capped with a tip complex [31,32,33,34,35] that is involved in sensing host cell contact to direct the recruitment of translocator proteins that coordinate to form a translocon pore in the host cytoplasmic membrane [36,37,38]. The initial subversion of host cell activities (the events leading to cellular invasion) appears to be initiated by the effector functions of the translocator proteins with invasion plasmid antigen C (IpaC) initiating actin mobilization and the early steps of membrane ruffling [39,40,41]. In some cell types, effector functions are also observed for the translocator protein IpaB (activator of caspase-1 for macrophages killing) and for the tip complex protein IpaD (caspase activation and mitochondrial damage in macrophages) [42,43]. It has also been suggested that IpaD is involved in targeting B cells (TLR2-mediated apoptosis) to dampen the host immune response [44].

## 3. The *Shigella* Injectisome: The Exposed Needle, Tip Complex and Translocon (Top)

### 3.1. The Needle

The *Shigella* and *Salmonella* T3SS injectisomes were first visualized a little more than 20 years ago after the complex was isolated from whole bacteria [38,45,46]. The two structures were remarkably similar and bore a striking resemblance to a needle and syringe. As the conservation of this nanomachine became evident among all the virulence T3SS studied, a uniform nomenclature was proposed and is now becoming routinely used (see Table 1) [7,47,48]. While the *Shigella* nomenclature will be used here due to the focus of this review, the universal nomenclature will be used when referring to proteins from non-*Shigella* systems. Ultimately, the surface exposed “needle” of the *Shigella* injectisome was found to be composed of a polymeric assembly of a small protein called MxiH [49]. The MxiH monomer is a small protein (83 amino acids) with a helix-turn-helix structure [30]. Such coiled-coil motifs appear to be common themes among the extracellular components of the injectisome. The needle extends outward from the bacterial outer-membrane and has an inner channel through which effectors pass during active secretion [49]. Approximately 120 copies of MxiH make up the needle assembly, which is limited to about 45–50 nm in length; however, overexpression of *mxiH* can give rise to needles that are much longer and which increase the efficiency of bacterial entry into cultured cells. In the absence of MxiH overexpression, the needle length is controlled by the regulator protein Spa32. Ironically, *spa32* null mutants possess needles up to ten times their normal length, but which do not support normal effector delivery for cellular invasion [50,51]. Spa32 is eventually secreted, triggering a switch in secretion from the needle protein to needle tip and translocator proteins [51].

The *Shigella* injectisome needle has an outside diameter of about 7 nm and an inner channel diameter of 2.5 nm with the MxiH monomers assembled in a helical manner with ~5.6 subunits per turn [49]. This assembly is similar to that observed for the helical assembly of the extracellular portions of the Gram-negative bacterial flagellum. It has been proposed that the packing of the needle could have a role in propagating secretion signals from the tip of the needle to the machinery below [30,52,53]. This altered packing would not be unlike the rearrangements seen in flagellar filament rotational switches. Indeed, specific amino acid changes in MxiH gives rise to phenotypes that suggest the *Shigella* T3SS needle is indeed implicated in having a role in controlling secretion with regard to the amount of protein exported and perhaps in the selection of secretion substrates. A similar phenomenon has been described for SctF from *Salmonella* and specific mutations in the *Yersinia* SctF needle can influence secretion control [54,55,56] and translocation [56].

**Figure 1:**
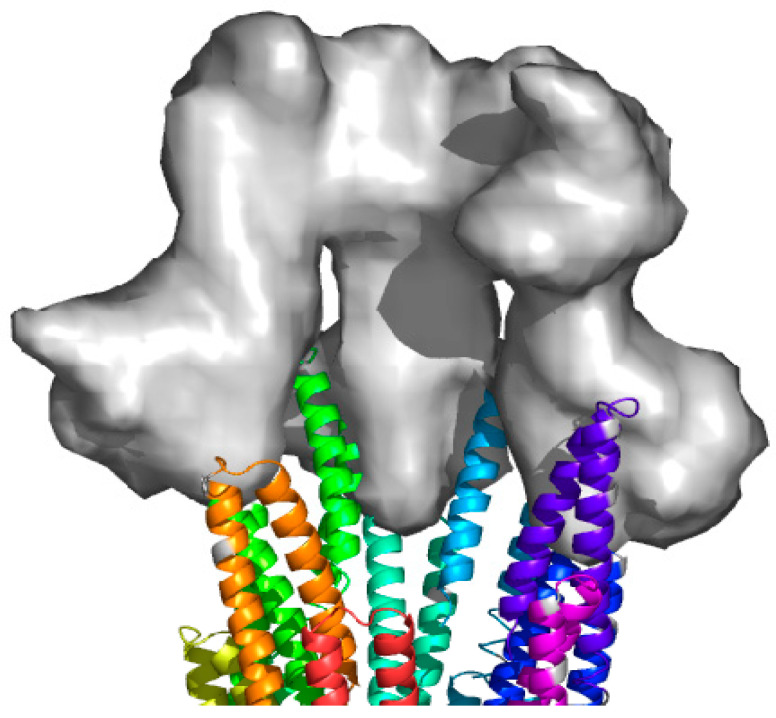
Model of the tip protein complex at top of the injectisome needle. A rendered model of three of the five subunits of the *Yersina* SctA tip complex protein is shown interfaced with an assembled model of the SctF needle based upon the crystal structure of MxiH (PDB-2CA5). Adapted with permission from [30], PNAS, 2006.

Indeed, it seems logical that the sensing of host cell contact, which would necessarily occur at the needle tip, would need to be sensed within the bacterial cytoplasm via signals that pass through the needle itself. How such signals would be received at the tip of the MxiH assembly was not clear during these earlier studies. However, clues were provided for the *Shigella* system by the observation that deletion of the genes for IpaD, the protein later identified as the needle tip complex protein (Figure 1), or IpaB, which was later identified as the first translocator protein, resulted in uncontrolled secretion [57,58,59].

### 3.2. The Needle Tip Complex Protein—IpaD

Invasion plasmid antigen D (IpaD) was identified as the injectisome needle tip protein (Figure 1) in 2006 after having been known for some time as being essential for control of type III secretion in *Shigella* [30,31]. IpaD and needle tip complex proteins from other systems display relatively little sequence homology; however, a common structural theme among them is that they possess a coiled-coil motif that provides structural stability [60]. Unlike the *Yersinia* SctA tip complex protein, IpaD (and the more closely related SctA proteins from *Burkholderia pseudomallei* and *S. typhimurium*) does not require cytoplasmic chaperones as part of its pathway to the injectisome needle tip [61,62]. It is possible that the lack of a cytoplasmic chaperone is related to the presence of an additional coiled-coil domain at the N-terminus of these tip proteins, which may provide a self-chaperoning function [60]. In the case of IpaD, it is possible that interactions between the central coiled-coil and the N-terminal domain may be displaced by interaction with the helix-turn-helix motif of the MxiH needle protein to anchor IpaD at the needle tip [63].

As mentioned above, IpaD is required for control of type III secretion in *Shigella* and this also appears to be the case for SctA in *Salmonella* [57,64,65,66,67]. IpaD’s ability to control secretion requires that it is stably bound at the tip of the needle [65] and this requires residues located at the C-terminus of the protein [65]. Because there is strong sequence conservation at their C-termini, it seems likely that this is true for *Salmonella* and *Burkholderia* SctA, as well. When originally described in *Yersinia*, the injectisome needle tip complex was proposed to consist of five copies of SctA [68]. For nascent *Shigella* injectisomes, IpaD has been described as the only protein present, presumably as a pentamer, at the tip of the *Shigella* injectisome needle [30,65]. However, when needles from *Shigella* overexpressing *mxiH* are mechanically sheared from the bacterium, IpaB can be detected by immunoblot analysis that is masked from surface detection using immunolabeling methods [31]. It should be noted that some groups suggest that IpaB may also be a component of the tip complex [32,34,67,69] and this is how it contributes to the control of type III secretion in *Shigella*. The roles of IpaB and IpaD as part of the tip complex include controlling secretion since the polar deletion of either results in uncontrolled and nonproductive type III secretion [57]. Nevertheless, based on the originally proposed SctA-containing tip complex from *Yersinia*, IpaD has been modeled at the *Shigella* needle tip as a pentamer (Figure 1) and this fits well with some reconstructions of the needle tip [70]. Furthermore, for wild-type *Shigella* the majority of complexes observed on the *Shigella* surface possess an IpaD pentamer at the tip and IpaD exists as a pentameric tip complex in *ipaB* null strains, indicating that there is no requirement for IpaB in forming the tip complex [34]. In contrast, an *ipaD* null mutant is unable to retain any IpaB on the *Shigella* surface [32]. Regardless of the precise timing, however, it is clear that at some point during the onset of secretion induction, IpaB must be able to interact with IpaD at the tip of the injectisome needle.

It has been observed that IpaB becomes easily detected at the *Shigella* needle tip when the bacteria are exposed for even short periods of time to bile salts [32,69,71]. IpaB recruitment is also observed when the bacteria are exposed to other factors such as serum-free tissue culture media (unpublished observation) and possibly factors that accumulate in the bacterial growth media in late exponential phase cultures. The role of IpaB as part of an intermediate or primed tip complex is discussed below. The presence of IpaB requires that IpaD already be present at the needle tip and, interestingly, while IpaD is retained at the needle tip in an *ipaB* null strain, that population of IpaD is rapidly lost from the needle tip when the bacteria are exposed to bile salts. While a strict consensus definition of the composition of the injectisome tip complex in *Shigella* remains to be established, it is clear that initiation of tip complex formation requires IpaD and very likely possesses an IpaD homopentameric intermediate.

### 3.3. Tip Complex Maturation: The First Step in Translon Formation

A hallmark of all the T3SSs examined to date is the generation of a translocon pore that is inserted into the host cell membrane to provide an entry point for subsequent effector proteins. Translocon pore formation requires multiple copies of two hydrophobic proteins that generate a membrane pore that remains associated with the tip complex protein (IpaD in *Shigella*). In *Shigella* it has been shown that the first translocator protein to be recruited to the needle tip is IpaB. While some groups have reported low levels of IpaB at the *Shigella* surface in the absence of external stimuli [32,34,67,69], bile salts have been shown to induce the recruitment of significant amounts of IpaB to the needle tip complex [32,69,71]. This event could be viewed as a priming of the T3SS since it is accompanied by a significant (five-fold or more) increase in *Shigella*’s ability to invade cultured cells [32]. IpaB recruitment is not accompanied by a significant increase in overall type III secretion or the recruitment of IpaC to the bacterial surface as would occur with secretion induction by small molecules such as Congo red. In fact, exposure to Congo red not only results in a complete onset of secretion induction, but it also causes a reduced ability for the bacteria to invade host cells [36].

IpaB is maintained in the *Shigella* cytoplasm as a complex with its cognate chaperone IpgC, which also serves as a chaperone for the second translocator protein IpaC [72]. The translocator–chaperone complex serves multiple roles in that it prevents the degradation of IpaB or IpaC prior to export, likely protects the bacterium from the potential membrane self-damage, and it sequesters IpgC which, once free of its translocator binding partner, is able to interact with the AraC family transcription regulator MxiE [73]. The translocator–chaperone complex exists as a heterodimer; however, free IpgC forms a homodimer that can associate with two copies of MxiE [74], after which this new complex becomes active as a transcription regulator. The MxiE-containing complexes bind to 17-bp MxiE boxes on the *Shigella* virulence plasmid to upregulate several late effector proteins [73]. In the presence of bile salts, the recruitment of IpaB to the bacterial surface is most likely not sufficient to trigger large changes in effector protein biosynthesis; however, it does appear to allow for the secretion of OspE1 and OspE2 to enhance the adherence of *Shigella* to the epithelial cell surface prior to actual invasion [75]. How bile salts induce signals to the bacterium for IpaB recruitment and/or OspE secretion is not entirely clear; however, bile salts bind directly to IpaD and induce conformational changes within the stabilizing central coiled-coil [76]. Once IpaB is mobilized to the injectisome needle tip, it is readily detected by immunofluorescence staining and immunogold labeling [32].

One of the difficulties in fully understanding the biochemistry of IpaB has been the poor solubility of this hydrophobic protein. It is readily purified as a soluble recombinant protein complex with IpgC; however, purifying it for structural analysis has not been possible [72]. Even crystallization of the highly soluble IpaB–IpgC complex has proven difficult. Initially, only small peptides from IpaB in complex with IpgC were described structurally [77], but more recently, a crystal structure was solved for 225 residue portion of AopB, the IpaB homologue from *Aeromonas hydrophila*, in association with its chaperone AcrH [78]. Nevertheless, biochemical study of the intact IpaB has been made possible by purifying the IpaB–IpgC complex and then removing the chaperone using mild detergents that maintain the protein’s structure and solubility in aqueous solution. Interestingly, the detergent used for preparing IpaB can have dramatic effects on its solution behavior while maintaining its structure [37].

Whether prepared in the zwitterionic detergent lauryl-N,N-dimethylamine oxide (LDAO) or the uncharged detergent n-octylpolyoxyethylene (OPOE), IpaB maintains a largely α-helical structure; however, other characteristics of the protein can differ significantly [37]. In OPOE, IpaB oligomerizes into a tetramer while in LDAO it forms a monomer. Both forms of the protein interact with phospholipid vesicles as determined by membrane flotation studies. In contrast, however, only the tetrameric form of IpaB is able to facilitate the release of small molecules from liposomes by forming structures within the phospholipid membrane that appear to be pores having a diameter in the range of 3–3.5 nm [37]. Based on this property, it has been proposed that when multiple copies of IpaB are recruited to the T3SS needle tip, they are primed for insertion into the host cell membranes where they are able to form pretranslocon pores. This would explain why *ipaC* null mutants that are unable to form the mature or complete translocon pore (visualized by complete contact-mediated hemolysis) are still able to mediate a low level of contact-mediated hemolysis activity (10–15% hemolysis) [39,79]. It has also been reported that large IpaB complexes can form ion channels in the host cell membranes and that this can lead to pyroptosis in macrophages [80]; however, those structures appear to be distinct from the ones described for the IpaB tetramers.

It has been difficult to assess thoroughly the structure–function relationships for IpaB in the absence of more detailed structural information. Initially, the only structure solved for IpaB has been for an N-terminal domain from residue 74 to 226 (with an additional 14 residues revealed in a subsequent crystal structure) that forms an elongated coiled-coil [81]. Despite only representing about 25% of the protein, this region appears to be important for a number of functions. This region is able to bind to the IpgC chaperone, inducing conformational changes involving regions outside of the coiled-coil [82]. It also acts as the putative anchor that maintains IpaB at the needle tip in association with IpaD [35]. Interestingly, the binding of this IpaB domain to IpaD only occurs in the presence of bile salts, possibly due to structural changes within IpaD caused by bile salt binding [35]. It has also been reported that mutations within this region can significantly reduce sensitivity to Congo red induction of type III secretion [83], possibly linking the interactions between IpaD and IpaB in secretion control.

Dissection of IpaB functions by mutagenesis has revealed important functional regions in the absence of a high-resolution structure. Deletions of short segments at the IpaB C-terminus suggest this region is involved in secretion control and Congo red sensitivity as measured by the induction of secretion [84,85]. Internally, 10 residue deletions suggest a similar role for a region between positions 227 and 306, along with residues 410–417 [84,85]. A complicating factor for such experiments is the inability to discern the structural effects caused by these deletion mutations. In a completely different mutagenesis strategy, the insertion of the bacteriophage T4 lysozyme (T4L) at predicted loops within IpaB was used to determine how added density can affect IpaB function [86]. T4L was used because this protein’s N- and C-termini are very close together, thereby minimizing the likelihood that the T4L insertion within loops will disrupt protein folding. The resulting chimeric proteins could all be purified as a stable complex with the IpgC chaperone with two exceptions. The mutants with T4L at positions 60 and 435 were not stable and degraded in the *Shigella* cytoplasm and this led to very high rates of uncontrolled secretion. It is likely that the presence of T4L at these positions blocks chaperone binding. This suggests that the C-terminal portion of IpaB, near residue 435, might also interact with the chaperone. Alternatively, T4L at residue 435 could prevent a major conformational change that is needed for chaperone binding. Either of these possibilities would be consistent with the fact that the IpaB–IpgC complex does not associate with phospholipid membranes or lyse liposomes. In all cases where T4L insertion does not negatively impact its purification with IpgC, IpaB can be separated from the complex and readily lyse liposomes. Perhaps the most intriguing data from this study was the observation that insertion of T4L at the end of the N-terminal coiled-coil (for which a structure has been solved) shows a complete loss of secretion control while retaining near wild-type levels of translocon formation (contact-mediated hemolysis) and invasion [86]. This suggests that control of secretion is not absolutely required for IpaB to carry out its invasion-related functions.

### 3.4. Completion of the Translocon and Induction of Secretion

Translocon formation requires input from a second translocator protein, which for *Shigella* is IpaC [87,88,89]. It has been shown in *Shigella* that IpaC is recruited to the needle tip upon bacterial contact with phospholipid membranes containing sphingolipids and cholesterol [36] and this signals the onset of secretion induction and translocon formation. Like IpaB, the study of IpaC is hampered by difficulties in purifying the protein. IpaC can be purified with IpgC as a homodimer and the two separated using mild detergents [90], but no successful attempts at determining a high resolution structure for any part of IpaC have been reported. IpaC is required for contact-mediated hemolysis, presumably due to its role in completing translocon pore formation [87]. Likewise, it is implicated in vacuolar escape [88,91] and it was reported to promote *Salmonella* entry into the cytoplasm when used in place of the *Salmonella* homologue SctB (SipC) [92]. This would suggest subtly different roles for *Salmonella* SctB and IpaC because *Shigella* with IpaC replaced by its *Salmonella* homologue appears to have a diminished ability to escape into the host cell cytoplasm.

IpaC (and *Salmonella* SctB) are not only believed to contribute to translocon pore formation, but also to have effector functions directly related to pathogen entry [39,92,93,94,95]. Both proteins have been implicated in the nucleation of actin, which at the site of injection into the cytoplasm produces membrane ruffles that are a hallmark of epithelial invasion by these pathogens. Furthermore, IpaC has been found to interact with the small GTPase Cdc42, which is implicated in *Shigella* entry into host cells [39]. The region of IpaC possessing its effector function lies at the immediate C-terminus of the protein where the deletion of 19 amino acids results in a complete loss of host cell invasion with no reduction in translocon formation as measured by contact-mediated hemolysis. Immediately upstream from this tail is a proposed coiled-coil structure whose existence was determined using linker-scanning mutagenesis. As with IpaB, the functional organization of IpaC has largely been determined using mutagenesis [96]. Thus, a great deal more remains to be learned of IpaC as more refined methods are applied to its study.

Like IpaB prepared in detergents, IpaC can interact with membranes and lyse liposomes to release their content [39,40,97]; however, attempts by our laboratory to demonstrate IpaC pore formation have proven unsuccessful (unpublished observation). This and the fact that once *Shigella* is stimulated to secrete IpaC its T3SS is fully activated with large amounts of IpaC secreted (along with the other Ipa proteins and then effector proteins), could indicate that IpaC’s role goes beyond that of acting as a translocon pore component [36]. It is possible that an important role for IpaC is to mechanically disrupt the *Shigella*-containing vacuole. Based on lipolysis studies with IpaC and genetic swapping experiments involving *Salmonella* SctB, a high concentration of IpaC is present in the vicinity of the translocon pore. This may cause localized membrane destabilization and facilitate rapid escape into the host cell cytoplasm. Certainly, the effector function of IpaC can be separated from its translocon formation capacity based on effects of C-terminal mutations on uptake while not affecting contact-mediated hemolysis [39]. The existence of an IpaB pretranslocon pore has been postulated based on the fact that *ipaC* null mutants that still have partial contact-mediated hemolytic activity. This could mean that the complete hemolysis seen for wild-type *Shigella* is facilitated not only by pore formation, but also by IpaC’s intrinsic membranolytic activity. Unfortunately, a model in which IpaC possesses detergentlike properties is not fully consistent with the osmoprotection studies where sufficiently large osmolytes can prevent contact-mediated hemolysis [38]. If IpaC truly behaved like a detergent in disrupting membranes, one might not expect osmoprotectants to protect against contact-hemolysis by wild-type *Shigella*. Nevertheless, the ability for IpaC to guide *Salmonella* vacuolar escape would fit with a “detergent” role for IpaC. Future work better addressing the biophysical properties of IpaC (versus SipC) should shed light on this problem.

The in situ translocon has been difficult to fully characterize at the molecular level due, in part, to its small size relative to the rest of the injectisome. A recent cryo-electron tomography study has revealed the general shape of the *Salmonella* translocon formed at the pathogen–host interface by the IpaD and IpaB homologues SctA and SctE, respectively [98]. A similar study targeted visualization of the pathogen-host–membrane contact site for the *Chlamydia* T3SS [99]; however, no such studies are yet available for the *Shigella* system. It is anticipated that future studies involving the *Shigella* system will provide improved insight into the roles and positions of IpaD, IpaB and IpaC within the translocon. Furthermore, the abundant mutant libraries available for all three of these proteins with associated phenotypic data should yield information on both the host and pathogen contributions to translocon assembly.

## 4. An Overview of the Envelope-Spanning Basal Body (Middle)

### 4.1. The Major Ring Components of the Basal Body

The stable injectisome needle complex (apparatus minus the more fragile sorting platform) consists of a needle (described above) that is embedded in a basal body structure that spans the bacterial inner (IM) and outer (OM) membranes [38,45,46]. The outside diameter of the OM ring portion of the basal body is 15 nm and this diameter increases to 25 nm as you progress downward to the IM ring with the overall height of the stacked rings of the basal body being 31 nm [100]. Conceptually, the basal body is a relatively simple structure composed of multiple rings with SctC (*Shigella* MxiD) forming the secretin at the OM and SctD/SctJ (*Shigella* MxiG/MxiJ) forming the IM ring [100,101,102]. The latter (IM ring) ultimately provides the anchor for the sorting platform in the functional injectisome [29,103]. Most of what we know structurally about these ring components comes from the *Salmonella* and *Shigella* systems. The organization of the SctC secretin ring remained a mystery for some time but it is now being revealed by high-resolution cryo-electron microscopy (cryo-EM) [101]. The secretin ring is assembled as a massive double-walled β-barrel structure within the OM with three N-terminal domains extending into the periplasm [101,104]. These domains contribute to gating of the secretin and are involved in formation the interface with the IM ring. For some time the circular symmetry of the secretin was unclear with estimates ranging from 12-fold to 15-fold. More recently, MxiD has been found to have a 15-fold symmetry [100]. In *Salmonella*, either a 14- or 15-fold symmetry for SctC was considered possible with 14-fold symmetry providing a better correlation with lower resolution cryo-EM density maps and the 15-fold symmetry showing better energy profiles in structural models [6]. When higher resolution cryo-EM maps were performed, however, a poorer correlation was seen for the 14-fold symmetry model, indicating that the 15-fold symmetry was indeed correct.

Beneath the β-barrel of the OM-imbedded ring are three important MxiD N-terminal domains, designated N0, N1 and N3 [100,101]. The connector region through which the secretin associates with the IM ring involves residues from 16 copies of N0 and N1 with the N0 domain actually making contact with the periplasmic domain of IM ring protein MxiG (called MxiG^P^ here—see below). The 16-fold symmetry here compensates for the seemingly odd symmetry mismatch between the secretin OM ring (15-fold symmetry) and the IM ring (24-fold symmetry) [100], which is similar to what has been observed in the flagellar system [105]. The origin of the sixteenth copy of the MxiD N-terminal domains N0 and N1 is unclear since no additional copy of the MxiD C-terminal domain has been identified within the structure. Within the connector region, two copies of N0 make contact with three copies of the C-terminal portion of MxiG to provide the connection interface [100]. Meanwhile, the OM ring portion of the MxiD ring possesses an inner channel of about 7 nm which is in accordance with the outer diameter of the MxiH needle. It is through this pore that the needle will ultimately assemble; however, until needle assembly occurs this opening remains closed by virtue of the gating provided by the extended loops of the MxiD N3 domain [101]. N3 has been shown to undergo substantial conformational changes between the open and closed gate. In the *Salmonella* system, two sets of β-strand pairs contribute to gating with these strands being bent to create the closed gate and extended and straight in the open gate. Hu et al. showed that a gradual repositioning of the *Salmonella* SctC secretin inner β-barrel strands 4 and 5 (GATE1) and β-strands 6 and 7 (GATE2) caused by the elongation of the needle from the inner rod gives rise to gate opening [101]. A similar process occurs for the MxiD secretin in *Shigella* as reported by Lunelli et al. [100].

On the side of the periplasm, opposite of the MxiD OM ring lies the IM ring which is formed by concentric inner and outer rings. The inner ring is formed by MxiJ (SctJ) in *Shigella*. MxiJ is a strictly periplasmic structure with two domains connected via a nine-residue linker [100]. MxiJ is held in place, at least in part, by its association with the outer ring component, which is generated from the protein MxiG (SctD). MxiG possesses a single transmembrane helix (residues 126 to 143) that connects the periplasmic domain (here designated MxiG^P^) with the MxiG cytoplasmic domain (MxiG^C^, residues 1–125), which has a noncanonical forkhead-associated domain structure [29,106,107,108,109]. The structure of MxiG^P^ has not been solved; however, the structure of its *Salmonella* homologue (SctD^P^) has [110] and based on the SctD^P^, structure the periplasmic portion of SctD has three domains, designated D2, D3, and D4. MxiG D2 and D4 are involved in MxiJ interactions and these interactions have been proposed to form solvent or ion channels that could participate in injectisome function [100]. Future work will be needed to determine whether this is indeed the case since many of the residues involved here are not conserved between the T3SS of different pathogens. The MxiJ and MxiG periplasmic domains all have similar folds known as the ring-building motif (RBM) [110]. The N3 domain of MxiD also has a RBM. In addition to interacting with MxiG^P^, MxiJ is palmitoylated at a cysteine near its C-terminus, which serves as an anchor to the IM [100].

### 4.2. The Accessory Components of the Injectisome Basal Body

While relatively large ring assemblies give rise to the overall architecture of the basal body consisting of MxiD, MixJ and MixG, the assembly and activity of a functional injectisome with external needle requires additional components. These components are required for initial IM ring assembly, stable association of the IM and OM rings, needle formation and injectisome function. The first step in assembly involves the insertion of the minor export apparatus proteins consisting of Spa24/Spa9/Spa29/Spa40 (sometimes referred to as SpaPQRS for both the *Salmonella* and *Shigella* systems [100] or SctRSTU using the unified nomenclature, respectively) into the IM [7]. Much of what we know of the export apparatus has been generated from work on the flagellar and virulence T3SS of *Salmonella* along with the T3SS of *Shigella*, so the universal nomenclature (Sct) [47,48] will be used here with occasional reference to the shared *Shigella*/*Salmonella* nomenclature. Like the basal body ring components, the membrane-associated export apparatus initially forms within the IM by sec-dependent secretion processes [111]. The process begins with the insertion of the minor components SctR, SctS and SctT (SpaPQR) into the inner membrane where they assemble into a transmembrane complex [4,7]. The assembled export apparatus is composed of five SctR (SpaP) and SctS (SpaQ) dimers are that are arranged in a helical manner to give a conical shape that is then topped with SctT (SpaR) [100]. As part of this complex, SctT has been suggested to resemble a SctR-SctS (SpaP-SpaQ) fusion [112]. It has been reported that the number of SctS (SpaQ) subunits can be variable to give either a stoichiometry of SctR_5_S_5_T_1_ (SpaP_5_Q_5_R_1_) or SctR_5_S_4_T_1_ (SpaP_5_Q_4_R_1_) within this assembly [112]. The assembly of the export apparatus is a prerequisite for the ensuing steps of assembly for an active injectisome. Interestingly, while the putative transmembrane domains of SctS (SpaQ) can be replaced with an alternative hydrophobic sequence that still allows injectisome assembly, such mutants cannot support active type III secretion [113]. This may be because SctS (SpaQ) has a role in the proper positioning of the export apparatus within the context of the dynamics of the secretion process. The SctV (*Shigella* MxiA) export gate is then recruited, but it is not entirely clear if this occurs before or after IM ring nucleation and assembly around the export apparatus [114,115].

Once formed in the IM, the export apparatus complex allows for nucleation of the MxiJ/MxiG IM ring via interactions with two loops from MxiJ [100]. This is initiated by interactions with SctJ (MxiJ) [4]. The more hydrophobic of the two loops of SctJ (MxiJ) may interact with SctR (SpaP) while the other interacts with SctS (SpaQ). It is at this point that the export apparatus is sequestered away from the IM itself in what could be described as a nanodisc structure [102]. Sequestration of the export apparatus into the nanodisclike environment is the result of membrane remodeling within the IM ring, as was demonstrated for the *Salmonella* system [116]. The SctC (MxiD) secretin ring forms separately in the OM after movement of this ring component into the periplasm by a sec-dependent process [7]. The two ring structures colocalize either concomitant with or perhaps following the association of the export gate formed by SctV (MxiA). As part of the basal body assembly process, SctU (SpaS) associates with the export apparatus, either via association with the export apparatus assembly or via association with the export gate formed by SctV (MxiA) [117]. Though best considered a component of the injectisome basal body, MxiA will be discussed in more detail below alongside the components of the sorting platform. The final export apparatus consists of the four minor components SctPQRS, which are located within the IM ring formed by SctJ and SctD (MxiJ and MixG, respectively, in *Shigella*) [7] and associated with the export gate formed by SctV (MxiA). It is at the outermost point of the export channel that the basal body inner rod (SctI or MxiI in *Shigella*) is then assembled [100].

It is at this point that the sorting platform (described in more detail below) can associate with the IM ring via the cytoplasmic domain of SctD (MxiG^C^), after which the type III secretion-dependent steps of assembly and effector protein export can commence [7]. As alluded to above, the first of these steps in the assembly process is the formation of the inner rod comprised of SctI (MxiI), which contributes to full basal body formation by assisting in the clearance of peptidoglycan, the recruitment of the SctC (MxiD) secretin, which provides a starting point for MxiH needle assembly [7,100,114]. Though additional mechanistic details remain to be worked out, the final basal body assembly in *Shigella* contains the large OM and IM rings (MxiD, MxiJ and MixG), the export apparatus (Spa24/9/29/40 or SpaPQRS), the export gate (MxiA) and the inner rod (MxiI). It is then from the inner rod that the MxiH needle extends and pushes open the gate provided by the MxiD secretin. At this point, the closed state of the inner channel is maintained by the export apparatus via the export gate (MxiA). Additional accessory proteins are ultimately needed for basal body formation or to generate a final functional injectisome. One of these proteins is the pilotin MxiM, which acts early in the assembly process by aiding in OM ring formation [118,119] after forming heterodimeric complexes with the secretin subunits within the periplasm [120]. Another key accessory protein is Spa32, which is responsible for controlling the substrate switch that controls needle length [50] by interacting with components of the sorting platform [121] and the export apparatus protein SctU [122]. It has also been reported that the multifunctional needle tip complex protein IpaD may be involved in substrate selection and switching via interactions with the export gate (MxiA) and secretin (MxiC) [123].

## 5. The Cytoplasmic Sorting Platform (Bottom)

### 5.1. The Sorting Platform Components

**Figure 2:**
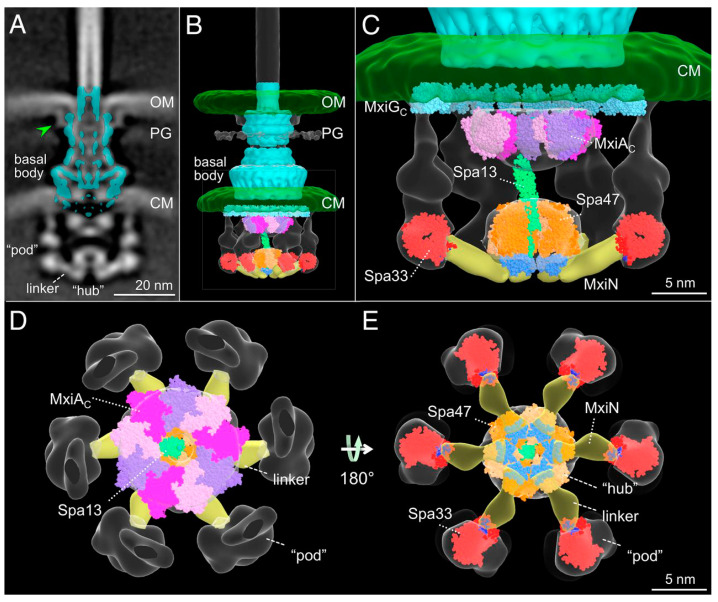
The first reported model of the in situ *Shigella* injectisome with sorting platform. (**A**) The isolated *Shigella* basal body (cyan) is fit onto the intact injectisome map and shown as a cross-section. The central portion of this cut-away of the core (cyan) contains the export apparatus (contained inside the inner membrane ring). Above this is the inner rod that eventually connects to the needle filament that crosses the outer membrane (OM). (**B**) A surface rendering of the isolated basal body is fit into the injectisome with the cytoplasmic portion of MxiG (SctD) and the sorting platform structures fit into the cytoplasmic densities. The extra densities shown include the OM, inner or cytoplasmic membrane (CM) peptidoglycan (PG), the sorting platform and an additional element (green arrow) near the OM. (**C**) Models of selected cytoplasmic proteins are fit into the rendering map. These include the cytoplasmic portion of the export gate (MxiAc, PDB-4A5P), Spa13, Spa47, MxiN and Spa33. (**D**) A top view of nonameric MxiAc with portions of the soring platform visible beneath. (**E**) A bottom view that more clearly shows the positions of Spa47, MxiN and Spa33. Structures used for this first model of the in situ *Shigella* injectisome include the *Thermus thermophilus* V-ATPase for Spa47 (orange, PDB-3J0J), *Salmonella* FliJ for Spa13 (green, PDB-3AJW), the cytoplasmic domain of *Salmonella* SctD for MxiGc (cyan, PDB-3J1W), the *Thermatoga maritima* FliN tetramer for Spa33 (red, PDB-1YAB). The MxiN density is shown in yellow with the interaction with Spa33 in blue and the interaction with Spa47 in cyan (**E**). Adapted with permission from [28], PNAS, 2015.

**Figure 3:**
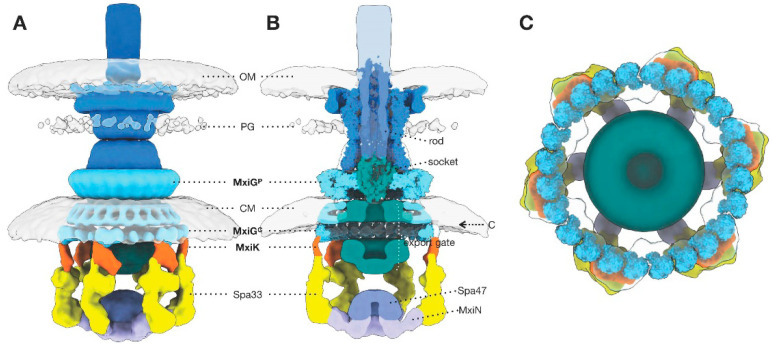
A surface rendering of the entire injectisome. (**A**) A rendering of the sorting platform shows the positions of the complete Spa33 pods (yellow), MxiK (orange), the cytoplasmic domain of MxiG (MxiG^C^) and its periplasmic domain (MxiG^P^) (cyan). The cytoplasmic membrane (CM), well wall (PG) and outer membrane (OM) are also indicated. (**B**) A cut-away of the structure in panel A allows visualization of the MxiA export gate density and the structures of *Salmonella* homologues of MxiG^C^ (PDB-3J1W) and MxiG^P^ (PDB-6DUZ). The export apparatus is represented by the flagellar FliPQR core (socket, PDB-62D). The secretin is represented by *Salmonella* SctC (PDB-6DV3) and the inner rod/needle filament (rod) is modeled from *Salmonella* SctF (rod PDB-6DWB). (**C**) A top view of the MxiG^C^ docking model (cyan) for the *Shigella* in situ injectisome is shown to directly overlie the Spa33 pods (yellow) which are associated with MxiG^C^ via the adaptor protein MxiK. Adapted from [29], American Society for Biochemistry and Molecular Biology Inc., 2019.

The sorting platform was proposed as a means for determining secretion substrate hierarchy in 2011 [124]. It was first actually visualized in 2015 when it was found to possess six large “pod” structures comprising multiple copies and forms of the protein Spa33 in *Shigella* (SctQ) (Figure 2 and below in Figure 3) [28,29]. Once visualized, it confirmed early observations that the cytoplasmic component of the *Shigella* injectisome was comprised of the Spa47 ATPase, Spa33, MxiN and MxiK [125] with Spa33 and Spa47 being the largest densities within the overall structure [28]. Because of the relatedness of the virulence T3SS with the flagellar T3SS, it was initially assumed that the dominant component of the sorting platform would be equivalent to the flagellar C ring [126,127]. This was consistent with the finding that this sorting platform component contained a large number of copies of SctQ (Spa33 in *Shigella*) [128]. The initial estimates were that there were 22 copies of SctQ in the *Yersinia* C ring [128].

When purified as a recombinant protein, Spa33 gives rise to a heterotrimeric structure containing one full-length copy of the protein and two smaller C-terminal fragments, Spa33^C-term^, generated from an internal ribosome-binding site present in the mRNA [127]. This also appears to be the case for other members of the SctQ family [129,130,131,132]. While the structure of full-length Spa33 has not yet been determined, the crystal structure of Spa33^C-term^ was solved and, based on the known structure of the homologous flagellar C-ring protein FliN from *Thermatoga maritima*, initially led to a model in which the main component of the sorting platform is a toroid structure [127]. The subsequent discovery of the podlike arrangement led to reconsideration of the structure–function relationships of the sorting platform (discussed below) [28]. There are proposed to be 22 to 24 copies of SctQ per sorting platform based on the data from the *Yersinia* and *Salmonella* systems, respectively [128,133]. This is not readily reconciled with the heterotrimeric nature of recombinant Spa33 [127] and its localization to six independent pods within the sorting platform [5,28].

It was reported by Lara-Tejero et al. that the alternative from of SctQ^C-term^ from *Salmonella* (equivalent to Spa33^C-term^) may actually not be present within the pods. Instead, SctQ^C-term^ in *Salmonella* is only needed for stabilization of sorting platform components during the assembly process [130]. This is not the case for Spa33 in *Shigella* [127] or SctQ in *Yersinia* for which SctQ^C-term^ was found to colocalize with assembled injectisomes [128]. One possible reason for this observation in *Salmonella* could be the low-level occurrence of the SctQ C-terminal domain even when the internal translation start site is removed as suggested by Bernal et al. [131]. Nevertheless, it does appear that the C-terminal variant is required to maintain the solubility of the full-length protein and for interactions involving other sorting platform components outside of the context of the sorting platform itself [130]. Interestingly, a report by Kadari et al. suggests that there are additional Spa33 variants and some may interact at other sites within the injectisome [132]. Based on the size of the Spa33 pods in the *Shigella* sorting platform, it is not clear that 24 copies of full-length Spa33 could be accommodated [28], which indirectly suggests that Spa33^C-term^ is a likely component of the pods. Likewise, it has been shown that elimination of the internal ribosome binding site abrogates type III secretion in *Shigella* [127] and this has been verified in our laboratory (unpublished data). It is clear that a great deal of work focused on the precise makeup of the sorting platform pods and how this makeup contributes to active type III secretion remains to be done.

The Spa33 pods are now known to associate with the protein MxiK (SctK) for which there are assumed to be six copies per sorting platform [29,103]. MxiK functions as an adaptor protein that interfaces the sorting platform with the cytoplasmic domain of the IM ring protein MxiG (SctD), which is referred to here as MxiG^C^ (SctD^C^) [29]. The MxiG^C^ (residues 1–125) structure was shown independently by NMR spectroscopy and X-ray crystallography to be that of a noncanonical forkhead-associated (FHA-like) domain [107,108]. FHA domains consist of an 11-strand β-sandwich possessing an “apical face” defined by loops located away from the N- and C-termini, and two lateral surfaces consisting of β-strands [5]. Canonical FHA domains are typically involved in phosphopeptide binding at the apical surface. Noncanonical “FHA-like” domains have been found that are involved in protein–protein interactions in a phosphopeptide-independent manner and this is the case for MxiG^C^ and its T3SS homologues [106]. In contrast, MxiK is rich in α-helical structure and is believed to form a novel protein fold based on the crystal structure of its SctK homologue from *Pseudomonas aeruginosa* (PscK) [103]. While MxiK has proven difficult to study as a purified recombinant protein, it can be purified as part of a complex when coexpressed with Spa33 (unpublished data). The importance of this complex in the dynamics of the sorting platform and its assembly remains to be fully established.

In addition to MxiK, the Spa33 pods also interact with MxiN (SctL), which forms radial “spokes” linking the pods with the Spa47 ATPase that forms a hub at the center of the sorting platform (Figure 2 and Figure 3) [28,29]. It has been shown that MxiN forms a homodimer using both two-hybrid analyses and analytical ultracentrifugation, thus suggesting that there are a total of 12 copies of MxiN within the sorting platform assembly [29,134]. The interaction of MxiN with the Spa33 heterotrimer can be demonstrated in vitro (unpublished data). Perhaps more importantly, MxiN’s interaction with Spa47 has been demonstrated in vitro and the dynamics of MxiN binding to different oligomeric states of the ATPase may have an important regulatory role in type III secretion [134]. No high-resolution structure has been solved for any member of the SctL (MxiN) family.

In contrast to MxiN, a crystal structure has been solved for the Spa47 ATPase and this was used to model a Spa47 hexamer for the identification of the key residues involved in the oligomerization and formation of the ATP hydrolysis active site [135]. Spa47 has significant sequence similarity to the catalytic moiety (β subunit) of the F1 ATP synthase [136,137]. The evidence provided to date suggest that it works in concert with other sorting platform components in the recognition of chaperones to promote the subsequent separation of the chaperone from its secretion substrate partner [138,139]. It then promotes the unfolding of the protein to be secreted and delivers it to the MxiA (SctV) export channel [138]. An important aspect of the role of Spa47 in type III secretion is its communication with the sorting platform protein MxiN, which serves as a regulator of the ATPase activity of the oligomeric form of Spa47 [134].

Another protein that interacts with Spa47 is the Spa13 (SctO) stalk protein, which links the ATPase with the MxiA export channel lying overhead (Figure 2 and Figure 3) [28]. The role of Spa13 in injectisome function is not entirely clear but work by Cherradi et al. suggests that it may bind chaperones, presumably in conjunction with Spa47, to help guide the secretion cargo to the MxiA export channel [140]. Spa13 was shown to bind the IpaB/IpaC chaperone IpgC as well as the export apparatus protein SctU [140]. The latter observation suggests that Spa13 may have a role in substrate switching during type III secretion.

### 5.2. Functional Features of the Sorting Platform

We are continuing to learn more about the assembly and structure of the sorting platform, aided by complementary molecular, biochemical, structural and imaging approaches. Even before visualization, the sorting platform complex was suggested to be instrumental in secretion–substrate selection and secretion hierarchy [124]. Nevertheless, there is still much to learn about the basic mechanisms that guide injectisome function. An attractive model put forth by Diepold et al. is that the sorting platform is a dynamic assembly with active secretion requiring an exchange of sorting platform components between the sorting platform and a cytoplasmic pool of these proteins [128]. For Spa33 (SctQ), the dynamic exchange between the sorting platform and cytoplasmic populations is a potential mechanism for guiding secretion cargo to the sorting platform for interaction with Spa47 and Spa13, followed by chaperone removal, unfolding of the cargo and cargo delivery to the export gate. The *Yersinia* T3SS has yielded seminal findings regarding a dynamic and adaptive network of cytoplasmic and sorting platform-associated proteins [141,142] and there is no reason to suspect that this does not hold true for other T3SS, including that of *Shigella*. The identification of soluble complexes of the *Salmonella* sorting platform proteins SpaO, SpaO^C^, OrgB and InvC (SctQ, SctQ^C^, SctL and SctN, respectively, or Spa33, Spa33^C^, MxiN and Spa47 in *Shigella*) is consistent with the stability of such cytoplasmic complexes [131].

Indeed, dynamic interactions are not only evident within the sorting platform but also at the interface between the sorting platform and the basal body. The cytoplasmic domain of SctD has been shown to adopt quite different conformations/positions within the active injectisomes of *Shigella* (MxiG^C^) and *Salmonella* (SctD^C^) [5,29]. The ability to adopt multiple positions is attributable to a linker that connects this FHA-like domain with the transmembrane helix that, in turn, links it to the periplasmic domain of the outer IM ring protein [107,108]. In *Salmonella*, SctD^C^ clusters into tetrads where contact is made with the sorting platform adaptor protein SctK [5]. This is not the case in *Shigella* where the adaptor protein MxiK interacts with MxiG^C^ as the latter forms an evenly spaced ring at the sorting platform–basal body interface [29] (Figure 3). Furthermore, in complexes that completely lack the sorting platform, the SctD cytoplasmic domain moves upward and inward to form a smaller ring structure that no longer lies directly over the positions that would be assumed by the sorting platforms pods in the active injectisome [29]. This level of flexibility suggests that there is the potential for communication between the SctD cytoplasmic domain and the export gate formed by MxiA. Interestingly, in the absence of the sorting platform, MxiA also shifts upwards and there is a closing of the channel within the basal body at both the OM secretin and within the IM ring-associated export apparatus (SctRSTU) [5,29].

The T3SS secretion process is believed to be driven by the proton-motive force (pmf), either harnessed via the MxiA (SctV) export gate [123] or perhaps by the IM ring proteins MxiJ/MxiG (SctJ/SctD) [100]. The involvement of MxiA is inferred from observations that FlhA represents the coupling point for the harnessing of the pmf by the flagellar T3SS (fT3SS) [143]. This activity is linked to a channel-associated loop (residues 145 to 208 in FlhA) with three residues being especially important (R147, D154 and D158). A similar loop with conserved charged residues is present within MxiA (residues 129–196), thus implicating this export gate protein in coupling the *Shigella* injectisome with the pmf. On the other hand, in high-resolution images generated by Lunelli et al., solvent accessible channels were identified in the IM ring made up of MxiG and MxiJ [100]. Mutations of charged residues within the visualized channel do have an impact on needle assembly, thus leading them to propose that electrostatic interactions here are also important for injectisome function. Between the complementary contributions made by the IM ring, the export gate and the sorting platform, a great deal of research remains in order to fully understand the mechanisms that guide type III secretion in *Shigella*. One aspect of this, the recruitment of cargo to the sorting platform, does appear to be governed by dynamic interactions within the sorting platform, but even these events require further clarification. Purified complexes consisting of SctQ, SctQ^C-term^, SctL and SctN from *Salmonella* have been prepared with low-resolution structures determined using small-angle X-ray scattering; however, the precise makeup and role of such structures and the stoichiometry of the components of such soluble complexes is unclear. Likewise, while it has been possible to trap secretion substrates within the injectisome [144], the visualization of the precise location of substrates within the sorting platform remains to be determined. It has been shown that chaperone–secretion substrate complexes can be shown to dock onto the export gate of the flagellar T3SS at the export gate (FlhA–equivalent to MxiA) [145]. Unfortunately, differences between the flagellar C ring and the pod structures of the virulence T3SS injectisome, along with the known dynamic nature of the sorting platform components of the latter makes it less than clear how such substrates would dock within the *Shigella* injectisome.

## 6. The *Shigella* T3SS as a Therapeutic Target

### 6.1. Targeting the Shigella T3SS with Small Molecule Anti-Infective Agents

As more bacterial pathogens, including *Shigella*, demonstrate emerging resistances to clinically important antibiotics, the search for new “anti-infective” agents has accelerated [146,147,148]. Such agents are proposed to block specific virulence factors to reduce the ability for a bacterial pathogen to cause pathology so that the body’s natural defense mechanisms can clear the infection. Such targeted inhibition is argued to be less likely to give rise to resistance because there are fewer selective pressures based on survival. Early studies on such inhibitors focused on the T3SS of *Yersinia* and *Pseudomonas,* which are closely related T3SSs [149,150]. Many of these inhibitors targeted transcription factors responsible for expression of important T3SS components, including effector proteins. For example, salicylidene acyl hydrazides have been shown to inhibit type III secretion in *Yersinia* [151], possibly by binding and inhibiting YopE effector activity [152], to inhibit the intracellular growth of *Chlamydia* [153], and prevent the secretion of some Type III proteins in *Salmonella* [154,155]. Other classes of inhibitory compounds have also been identified; however, most have not been well-characterized with respect to intracellular target and in many cases their inhibitory concentrations are well above what would be pharmacologically feasible. Many of the known anti-infective agents that target the T3SS apparatus have been best characterized in *P. aeruginosa*. In *Pseudomonas*, thiazolidinones were shown to target the SctC secretin, hydroxyquinolines were found to target the SctN ATPase, and phenoxy-acetamides were found to target the SctF needle [150]. In the same respect, SctA-specific antibodies were found to target the *P. aeruginosa* tip complex to reduce effector secretion and are potentially effective in animal models [156,157,158]. Unfortunately, not all compounds work equally well against all T3SSs and many of the compounds examined thus far have been tested in a limited number of systems, including the closely related *P. aeruginosa* and *Yersinia* systems. Thus, a relatively limited number of studies have explored compounds that would target the *Shigella* T3SS.

In one report focusing on the *Shigella* system, small molecules were screened for their ability to block *Shigella* type III secretion, to reduce the secretion of effector proteins after Congo red induction, and inhibition of secretion by mutants that constitutively secrete effector proteins. They were then examined for interference with *Shigella* T3SS-related virulence activities including macrophage apoptosis and bacterial entry into HeLa cells [159]. The compounds identified did not appear to result in a reduction in the levels of T3SS components such as MxiJ (IM ring) or MxiH (needle monomer), suggesting that they act on injectisome assembly rather than at the level of gene expression. Indeed, there did appear to be a compound-induced loss of needle formation on the *Shigella* surface, despite an increase in the apparent number of T3SS basal bodies seen on these bacteria [159]. Interestingly, rather than a complete loss of needle formation, there is a tendency toward “short” needles and this was determined to occur at the step of needle assembly. Despite the novelty of this finding in the *Shigella* system, the clinical relevance of such compounds may be compromised by the fact that such compounds might be more effective as a prophylactic measure rather than as a postinfection therapy since they do not appear to dramatically affect needles that have already been assembled [159]. On the other hand, the replication and spread of *Shigella* within and between mucosal epithelial cells would require the continual replenishment of injectisomes, which could render them vulnerable to these agents as long as their intracellular localization did not compromise the agent’s bioavailability during active shigellosis.

In contrast, a more recent study used the crystal structure of Spa47 (SctN) to identify T3SS-specific ATPase inhibitors [160]. Using three small molecules previously shown to inhibit YscN (SctN) from *Yersinia*, Case et al. used in silico docking to show that these compounds had the potential to bind at two distinct sites at the interface between subunits of the Spa47 hexamer. One site was located within the ATPase active site, with the second site about 2.5 nm away. The compounds were then tested for inhibition of ATPase activity in vitro and found to be noncompetitive inhibitors, suggesting they are not competing for ATP binding [160]. This was deemed an attractive feature since it suggests that there is less likelihood of interactions with other “off-target” ATPases. Furthermore, none of the compounds disrupted Spa47 oligomerization. These compounds had a minimal impact on *Shigella* growth and injectisome assembly, but prevented type III secretion in whole cells. While such inhibitors still must be used at micromolar concentrations to prevent type III secretion, they do show that it is possible to target the T3SS postassembly and thus have anti-infective potential against shigellosis. A compelling feature of these targeted compounds is their ability to enter bacterial and host cells with at most a modest effect on the *Shigella* or host cell viability or growth.

As previously mentioned, exposure to the antibodies generated against the *P. aeruginosa* needle tip complex protein SctA can serve as a potential biological anti-infective agent [156,157,158]. Similarly, IgG prepared against peptides derived from IpaD were shown to neutralize *Shigella* contact-mediated hemolysis activity [31]. More recently, it was shown that camelid antibodies can be generated against IpaD and expressed in *E. coli* as single-chain VHH domains. From a panel of anti-IpaD VHH domains, three were found to neutralize *Shigella* contact-mediated hemolysis to varying degrees [161]. Neutralization was greatest when all three were genetically fused. X-ray structures of IpaD-VHH complexes showed that the neutralizing epitopes are located in the central coiled-coil of IpaD and in what has been termed the “distal domain” [161]. It is possible that neutralization by these VHH domains is possible due to locking IpaD into a conformational state that is not compatible with tip complex maturation via the recruitment of IpaB to the needle tip, but this remains to be established.

### 6.2. Prophylactic Shigella Vaccines Targeting the Injectisome

Disease prevention by vaccination represents perhaps one of the greatest achievements of biomedical science. Vaccines against viral and bacterial pathogens have saved countless lives around the world. The Steering Committee on Diarrhoeal Disease Vaccines of the World Health Organization placed *Shigella* first on its list of priorities in 1966 as part of its Global Programme for Vaccines and Immunization [162]. Although a number of vaccine candidates and platforms have been considered for preventing shigellosis, no vaccine is yet commercially available. Such a vaccine would have its greatest outcome in developing regions of the world. Thus the WHO and other groups with an eye on global public health initiatives (e.g., the Bill and Melinda Gates Foundation and PATH) have put major efforts into the creation of vaccines that will reduce the childhood deaths and disabilities caused by *Shigella* and the Enterotoxigenic *E. coli* (ETEC).

Formal and coworkers were prominent figures in the early attempts to generate live, attenuated vaccines against *Shigella* in the 1960s [163]. Among these early studies were strains that became spontaneously avirulent (most likely through loss of the virulence plasmid) and strains that could invade but not maintain themselves in the lamina propria [163]. The latter were generated by mating *S. flexneri* 2a with *Escherichia coli*. Much later, *Shigella* auxotrophs were generated by transducing *Shigella* with a transposon-inactivated *aroD* gene, which allowed the pathogen to retain virulence functions in tissue culture models but did not cause disease in animal models [164]. The further development of live, attenuated *Shigella* vaccines continues today with combinations of auxotrophic mutations (*guaAB*) with the deletion of targeted virulence factors such as IcsA/VirG and the two *Shigella* enterotoxins (*sen* and *set*) [165]. Though none of these vaccines have been licensed for human use, they have advanced far enough to now include vaccine candidates such as the CVD multivalent strains that may be able to target both *Shigella* and Enterotoxigenic *E. coli* [166]. In addition to live, attenuated vaccines, there have also been forays into the potential use of inactivated *Shigella* vaccines, with one recent study describing the generation of a trivalent whole-cell vaccine that targets *S. flexneri* 2a, *S. sonnei* and *S. flexneri* 3a [167]. Many of these and other vaccines are reviewed as part of the ongoing international conference proceeding Vaccines Against *Shigella* and ETEC (VASE) for which useful summaries are regularly published [168].

A vexing problem with the development of *Shigella* vaccines is that the shigellae collectively have more than 50 somatic antigen serotypes and natural infection tends to target these abundant OM antigens [169]. Likewise, the inactivated *Shigella* vaccines and live-attenuated vaccines mentioned above elicit immune responses that favor targeting the most abundant available antigen, which is the surface-exposed LPS [167,169,170], which necessitates the creative generation of multivalent formats. The first subunit vaccines against *Shigella* included conjugates of *Shigella* O antigens with recombinant exoprotein A (rEPA) which was initially developed in Israel [171] and is being developed to recognize multiple serotypes using synthetic carbohydrate species [172]. Another approach has involved subunitlike vaccines targeting the *Shigella* T3SS that were generated from soluble extracts of the bacteria that contained large amounts of LPS and significant quantities of several different proteins, including the tip complex and translocator proteins IpaB, IpaC and IpaD [173]. These vaccines evolved to become what is now known as the Invaplex vaccine. A synthetic Invaplex containing *Shigella*-derived LPS and recombinant IpaB and IpaC has shown promise as a clinically relevant vaccine [174]; however, there is a potential for reactogenicity for this LPS-rich “subunit” vaccine and the resulting immune response is strongly directed toward the serotype-specific LPS rather than the highly conserved Ipa proteins [173].

Following discovery that IpaD is the needle tip complex protein and the finding that IpaB joins IpaD at the tip of the primed T3SS needle (in the presence of bile salts and some other small molecule stimulators), these proteins were considered prime candidates for a subunit vaccine in an LPS-free platform. Indeed, vaccination with IpaB and IpaD successfully protects mice from lethal challenge in the mouse lung infection model [175,176]. The protection was optimal when the proteins were delivered intranasally with the experimental mucosal adjuvant dmLT (double-mutant heat-labile enterotoxin from ETEC) [175,177]. The improvement of vaccine efficacy was observed when IpaB and IpaD were genetically fused (giving what has been coined the DB Fusion or DBF vaccine) [178]. The most attractive feature of the DBF vaccine has been its ability to show protection across serotype and/or species boundaries [178].

A shortcoming with regard to developing vaccines against *Shigella* is the absence of a good animal model of the infection. This is beginning to be addressed through the generation of human enteroid models that are now being used to confirm what we know about the pathogenesis of shigellosis [21,179]. As such systems are developed that include cells important for mediating protective immune responses, it is anticipated that our understanding of the cell biology of *Shigella* pathogenesis in humans will be improved and this will further aid in the development of a protective *Shigella* vaccine. The development of a serotype-independent vaccine that targets the *Shigella* T3SS would also have implications for generating vaccines against a significant number of T3SS-containing bacterial pathogens that are important public health problems globally.

## 7. Looking to the Future

The bacterial type III secretion system is an exquisite nanomachine that has been the focus of research for more than 20 years. With visualization of the needle complex came a wealth of new research on the assembly of this structure along with targeted research on the components of the translocator proteins, the needle itself and the structure–function features of the proteins making up the core complex or basal body. In recent years, increasingly higher resolution imaging of the entire injectisome and sorting platform has yielded a flurry of reports that are helping to build an atomic-resolution model of the entire apparatus as well as allowing dissection of the molecular dynamics that underlie the mechanisms governing type III secretion. However, much work remains to be done regarding the detailed structure of this assembly and the mechanisms that underlie type III secretion.

While a great deal is now known about the *Shigella* T3SS needle and tip complex, the structure of the translocon and how it contributes to the control of secretion induction remain unknown. An important step forward here would be a better understanding of the in situ tip complex for the nascent injectisome, the primed injectisome and the tip complex in association with the membrane-inserted translocon. In conjunction with routine molecular biology approaches, newly high-resolution imaging methods are evolving that will allow us to make valuable inroads here. A potential outcome of an improved understanding of the translocon will be the identification of targets for future drug intervention and immunotherapies. While such therapies might have limited use against shigellosis, a proof of principle demonstration of these in the *Shigella* system could have tremendous translational application to other pathogens that rely upon the T3SS for initiating human infections.

The core or basal body of the *Shigella* injectisome, while conceptually simple in its concentric ring composition, is now revealing the complicated dynamic nature of the steps that guide injectisome assembly. This is made even more interesting by emerging work on the interactions that occur between the relatively stable basal body and what is probably a highly dynamic sorting platform. The T3SS has established elegant gating scenarios that ensure the integrity of the bacterial envelop during basal body assembly and needle generation. At the same time, the gating mechanisms within the export gate ensure closure in the absence of a sorting platform that governs the selection, unfolding, and delivery of export cargo into the injectisome channel. The molecular basis for sorting platform function and the energetic drivers for the entire process, whether via the distinct contributions of the Spa47 (SctN) ATPase or the proton motive force, will be a major research focus for the foreseeable future. Once again, such research will reveal targets for new experimental therapies targeting the T3SS. We have learned a great deal about the T3SS injectisome and type III secretion in general from work conducted on the *Shigella* system. Moving forward, the comparative study of the related *Shigella* and *Salmonella* systems, in conjunction with parallel studies on other T3SS families such as the *Yersinia*/*Pseudomonas* family will yield further insight of the structural, molecular and physical basis for the T3SS.

## Figures and Tables

**Table 1:** Nomenclature for the *Shigella* Type III Secretion System Components

Subassembly Location	*Shigella*	*Salmonella*	Unified ^a^	Function/Location
Needle	MxiH	PrgI	SctF	Needle monomer
Tip Complex	IpaD	SipD	SctA	Tip complex
Translocon	IpaB	SipB	SctE	First translocator and tip complex protein in *Shigella*
	IpaC	SipC	SctB	Second translocator
Basal Body Rings	MxiD	InvG	SctC	Secretin
	MxiJ	PrgK	SctJ	Inner IM ring protein
	MxiG	PrgH	SctD	Outer IM ring protein
	MxiI	PrgJ	SctI	Inner rod
Basal Body	Spa24	SpaP	SctR	Export apparatus
Accessory Proteins	Spa9	SpaQ	SctS	Export apparatus
	Spa29	SpaR	SctT	Export apparatus
	Spa40	SpaS	SctU	Export apparatus
	MxiA	InvA	SctV	Export apparatus
	MxiC	InvE	SctW	Gatekeeper
	Spa32	InvJ	SctP	Needle length control
Sorting Platform	MxiK	OrgA	SctK	Adaptor protein
	Spa33	SpaO	SctQ	Pod protein(s)
	MxiN	OrgB	SctL	Radial spoke protein
	Spa47	InvC	SctN	ATPase
	Spa13	InvI	SctO	Stalk protein

^a^ The unified nomenclature is based largely on the Yersinia T3SS nomenclature [7,47,48].

## Data Availability

Not applicable.
